# Effectiveness and Tolerability of Oral Amoxicillin in Pregnant Women with Active Syphilis, Japan, 2010–2018

**DOI:** 10.3201/eid2606.191300

**Published:** 2020-06

**Authors:** Takeshi Nishijima, Kei Kawana, Ichio Fukasawa, Naoko Ishikawa, Melanie M. Taylor, Hiroshige Mikamo, Kiyoko Kato, Jo Kitawaki, Tomoyuki Fujii, Women’s Health Care Committee, Japan Society of Obstetrics and Gynecology

**Affiliations:** National Center for Global Health and Medicine, Tokyo, Japan (T. Nishijima);; World Health Organization Regional Office of the Western Pacific, Manila, the Philippines (T. Nishijima, N. Ishikawa);; Nihon University School of Medicine, Tokyo, Japan (K. Kawana);; Dokkyo Medical University, Tochigi, Japan (I. Fukasawa);; World Health Organization, Geneva, Switzerland (M.M. Taylor);; Centers for Disease Control and Prevention, Atlanta, Georgia, USA (M.M. Taylor);; Aichi Medical University, Aichi, Japan (H. Mikamo);; Kyushu University Graduate School of Medical Sciences, Fukuoka, Japan (K. Kato);; Graduate School of Medical Science, Kyoto Prefectural University of Medicine, Kyoto, Japan (J. Kitawaki);; Graduate School of Medicine, The University of Tokyo, Tokyo, Japan (T. Fujii)

**Keywords:** congenital syphilis, adverse birth outcomes, mother-to-child transmission, Treponema pallidum, amoxicillin, Japan, nationwide multicenter study, effectiveness, tolerability, safety, ampicillin, oral penicillin, pregnancy, active syphilis, prevention, alternative regimen, bacteria, sexually transmitted infection

## Abstract

We conducted a nationwide retrospective study in Japan to evaluate the effectiveness of oral amoxicillin or ampicillin as alternatives to injectable benzathine penicillin G for treating pregnant women with syphilis and preventing congenital syphilis (CS). We investigated 80 pregnant women with active syphilis treated with amoxicillin or ampicillin during 2010–2018. Overall, 21% (15/71) had pregnancies resulting in CS cases, and 3.8% (3/80) changed therapies because of side effects. Among 26 patients with early syphilis, no CS cases occurred, but among 45 with late syphilis, 15 (33%) CS cases occurred. Among 57 patients who started treatment >60 days before delivery, 8 (14%) had CS pregnancy outcomes. We found oral amoxicillin potentially ineffective for preventing CS cases among pregnant women with late syphilis but potentially effective in those with early syphilis. Prospective studies are needed to definitively evaluate the efficacy of amoxicillin for the treatment of pregnant women with syphilis to prevent CS.

Syphilis is a sexually transmitted infection that can be passed from mother to infant during pregnancy and childbirth. Mother-to-child transmission (MTCT) of syphilis results in congenital syphilis (CS), which can cause serious outcomes, including miscarriage, stillbirth, neonatal death, preterm birth, low birth weight, and various illnesses and congenital deformities. The World Health Organization (WHO) estimated 988,000 active syphilis cases and 611,000 CS cases in pregnant women worldwide in 2016 (*1*), and syphilis is the second most common infectious cause of stillbirth worldwide (*2*).

Injectable benzathine penicillin G (BPG) is the only regimen recommended in WHO and US Centers for Disease Control and Prevention (CDC) guidelines (*3*–*5*) for the treatment of syphilis in pregnant women to prevent CS. Sufficient evidence is not available to recommend an alternative regimen. In a systematic review on alternative treatments for pregnant women with syphilis, only 21 pregnant women treated with regimens other than BPG could be identified (*6*). Erythromycin and azithromycin do not cross the placental barrier and thus cannot treat infections in the fetus (*6*). Macrolide-resistant *T. pallidum* has been reported in many countries (*7*). Tetracycline and doxycycline are contraindicated in the second and third trimesters of pregnancy, and data are not sufficient to recommend ceftriaxone for treatment of maternal syphilis and prevention of CS (*4*,*8*). During 2014–2016, shortages or stockouts of BPG were reported by 39 of 95 surveyed countries and territories (*9*), and in several countries, including Japan, intramuscular BPG is not available (*10*). A reliable alternative treatment for syphilis in pregnant women to prevent CS is urgently needed.

Because sales of BPG stopped in Japan in 1986, oral penicillins, such as amoxicillin or ampicillin, have been primarily used to treat pregnant women with syphilis. These regimens are indicated in the obstetrics and gynecology care guidelines of Japan (*11*), which follows the regimens recommended by the Japanese Society for Sexually Transmitted Infections diagnosis and treatment guidelines (*12*). These guidelines recommend oral amoxicillin or ampicillin with dosing of 1,500 mg/day (i.e., 500 mg 3×/d) for 2–4 weeks for primary syphilis, 4–8 weeks for secondary syphilis, and 8–12 weeks for tertiary or later-stage syphilis in pregnant women. However, effectiveness of this regimen for pregnant women and prevention of CS has not been reported, except for 1 pregnant woman who was treated with a much higher dosage of amoxicillin (6 g/d) plus probenecid (1 g/d) for 14 days (*13*). In this retrospective study, we investigated the effectiveness and tolerability of oral amoxicillin and ampicillin in pregnant women to treat active syphilis and prevent MTCT of syphilis.

## Methods

### Study Setting and Population

We conducted this nationwide, multicenter retrospective study in Japan as a joint research project between the Japan Society of Obstetrics and Gynecology and the WHO (Appendix) (*14*). The study was approved by the human research ethics committees of Nihon University School of Medicine (Tokyo, Japan), Dokkyo Medical University (Tochigi, Japan), and the Japan Society of Obstetrics and Gynecology (Tokyo, Japan). We also submitted the study protocol to the WHO Ethics Review Committee. Because our research involved anonymized patient data and not new data collection or an additional intervention, the study was determined to be exempt from WHO Ethics Review Committee review. The requirement for informed consent was waived because this study included only data gained from routine clinical practice. We conducted this study according to the principles expressed in the Declaration of Helsinki.

We included pregnant women with syphilis, regardless of their symptoms, who were treated with oral amoxicillin or ampicillin during 2010–2018; we only included those with serum rapid plasma reagin (RPR) titers >8 and positive results from treponemal tests, such as the *T. pallidum* hemagglutination test (*10*,*15*). We excluded patients with tertiary syphilis or neurosyphilis diagnoses that were based on findings from cerebrospinal fluid (CSF) samples (*10*,*16*) or patient symptoms (including ocular or auditory syphilis) and those with suspected reinfections after initiation of syphilis treatment (defined as patients with >4-fold rise in RPR titer after 4-fold decrement with or without symptoms) (*10*,*15*,*17*).

In Japan, the automated latex turbidimetric immunoassay and the conventional manual RPR card test are both used to determine RPR titers (*18*,*19*). The automated RPR test highly correlates with the manual test for syphilis diagnoses (*18*,*19*); just like the manual card test, for the automated test, a 4-fold decrement in RPR titer is a good criterion for successful treatment of syphilis (*19*). Thus, we treated the titers from the automated RPR test the same as those from the manual RPR test.

### Definitions

We defined CS following the reporting criteria set by the Japanese Ministry of Health, Labour, and Welfare (*20*). Diagnosis of CS in newborns required fulfillment of any 1 of the following criteria: serum RPR titer >4-fold that of the mother’s (*4*); fluorescent treponemal antibody absorption (FTA-ABS) test result positive for serum IgM (*12*,*21*,*22*); lesion tissue or body fluid samples positive by PCR (*4*); lesion or fluid samples positive for *T. pallidum* on dark field microscopy (*4*); or physical examination findings consistent with CS, such as nonimmune hydrops, jaundice, hepatosplenomegaly, skin rash, pseudoparalysis, and rhinitis (*4*,*20*,*23*). We classified the mother’s stage of syphilis into early syphilis (primary, secondary, or early latent syphilis) and late syphilis (late latent syphilis or latent syphilis of unknown duration). We defined early latent syphilis as asymptomatic syphilis that could be linked to reported syphilis symptoms, a sexual exposure, or conversion from a prior negative syphilis test <1 year after diagnosis. These criteria occurring >1 year after diagnosis constituted a late latent case and at an undefined time point after diagnosis as a latent case of unknown duration (*4*,*10*,*24*). We defined miscarriage as the loss of a fetus before week 20 of pregnancy and stillbirth as the loss during or after week 20.

### Data Collection, Outcome Measures, and Statistical Analysis

We collected data from medical charts at each facility, using a case report form (Appendix). The primary outcome of this study was effectiveness of prevention of MTCT of syphilis. We defined MTCT as a CS case and defined CS cases as live newborns with CS diagnoses, miscarriages, or still births. If a live newborn was not given a CS diagnosis, we interpreted the treatment given as successful for preventing MTCT (*5*). We evaluated the secondary outcome serologic effectiveness of treatment in the mother (4-fold decrement of serum RPR titer) at each of the following time points: delivery (*25*), 6 months after therapy (*4*), and 12 months after therapy (*4*). We analyzed the primary outcome among all study patients and among those who initiated treatment >60 days before delivery. We used this subgroup to evaluate drug effectiveness among patients who initiated treatment within an adequate interval (defined by WHO as >30 days before delivery) (*26*). We used 60 days for this evaluation because of the long treatment duration recommended for oral penicillins in Japan’s guidelines (*12*). We compared the percentage of CS cases between those who had early syphilis and late syphilis, between those treated with amoxicillin and those treated with ampicillin, and between those who were and were not Japanese.

We compared characteristics between groups using the Student *t*-test for continuous variables and using either the χ^2^ test or Fisher exact test for categorical variables. We defined statistical significance as a 2-sided p value <0.05. We developed a multivariate logistic regression model to determine the effect of factors on CS cases. We performed this analysis with SAS version 9.4 (https://www.sas.com) and all other statistical analyses with SPSS Statistics 23 (https://www.ibm.com).

## Results

Of 88 hospitals invited into the study, 44 (50%) participated and provided 131 case report forms (Appendix Figure). We excluded 51 cases, resulting in 80 cases being included in our analysis.

Median patient age was 23 (interquartile range [IQR] 21–27) years; 75 (94%) patients were Japanese, and only 1 patient was HIV positive (Table 1). In total, 31 patients had early syphilis (5 primary, 19 secondary, 7 early latent) and 49 had late syphilis (4 latent, 45 latent with unknown duration). Median RPR titer at diagnosis was 51 (IQR 29–72), and 66 (83%) patients were treated with amoxicillin and 14 (17%) with ampicillin. The median duration of treatment for pregnant women was 60 (IQR 29–90) days, and the median duration of treatment before delivery was 56 (28–86) days. In total, 61 (76%) patients were treated with 1,500 mg/day dosing, and 3 were concurrently prescribed probenecid. Median gestational age of fetus at CS diagnosis was 13.8 (IQR 11.7–26.3) weeks and at treatment initiation 15.8 (13.0–27.1) weeks. In total, 57 (71%) patients received syphilis diagnoses when the fetus had the gestational age of <20 weeks. A comparison of pregnant women by syphilis stage revealed that women with late syphilis and early syphilis had similar characteristics but women with late syphilis had longer durations of treatment (Table 2).

**Table 1:** Characteristics of pregnant women with active syphilis treated with amoxicillin or ampicillin, by CS birth outcome, Japan, 2010–2018*

**Characteristic**	**All cases, n = 80**	**CS cases,† n = 15**	**Cases of live birth without CS, n = 56‡**	**p value§**
Age, y	23 (21–27)	24 (22–26)	26 (22–29.5)	0.46
Japanese	75/80 (94)	12/15 (80)	54/56 (96)	0.06
HIV co-infection	1/78 (1)	0/15	1/56 (2)	1.00
Syphilis stage				
Early syphilis	31/80 (39)	0/15	26/56 (46)	
Primary	5/80 (6)	0/15	5/56 (9)	
Secondary	19/80 (24)	0/15	14/56 (25)	
Early latent	7/80 (9)	0/15	7/56 (13)	
Late syphilis	49/80 (61)	15/15 (100)	30/56 (54)	
Late latent	4/80 (5)	0/15	4/56 (7)	
Latent with unknown duration	45/80 (56)	15/15 (100)	26/56 (46)	
Diagnosis and treatment				
Rapid plasma reagin titer at diagnosis	51 (29–72)	58 (28–105)	51 (27.1–71.8)	0.59
Amoxicillin	66/80 (82.5)	11/15 (73)	47/56 (84)	0.45
Ampicillin	14/80 (17.5)	4/15 (27)	9/56 (16)	
Antimicrobial drug dosage, mg/d	1,500 (1,500–1,500)	1,500 (1,500–1,500)	1,500 (1,500–1,500)	0.88
Received 1,500 mg/d	61/80 (76)	13/15 (87)	39/56 (70)	0.20
Co-administered probenecid	3/75 (4)	1/15 (7)	2/56 (4)	0.51
Total duration of treatment, d¶	60 (29–90)	70 (37–101)	56 (28–90)	0.49
Duration of treatment at delivery, d¶	56 (28–86)	68 (4–100)	56 (28–84)	0.84
Gestational age at diagnosis, wk¶	13.8 (11.7–26.3)	18.1 (12.3–34)	13.2 (11.5–21.6)	0.082
Gestational age at treatment, wk¶	15.8 (13.0–27.1)	18.3 (13.6–34.3)	15.9 (13.4–25.4)	0.22
Gestational age at delivery, wk¶	39.1 (37.9–40.6)	38.6 (35.6–40.9)	39.1 (37.9–40.3)	0.38
Time from treatment to delivery, wk¶	21.9 (8.5–26.3)	15.7 (0.4–25.9)	23 (16.7–27.2)	0.026
Started treatment <60 d before delivery¶	14/69 (20)	6/14 (43)	8/56 (14)	0.027
Birth outcomes				
Birth weight, g¶	2,936 (2,580–3,156)	2,704 (1,797–3,085)	2,959 (2,641–3,180)	0.094
Low birth weight, <2,500 g	11/69 (16)	4/13 (31)	7/56 (13)	0.20
Very low birth weight, <1,500 g	3/69 (4)	3/13 (23)	0/56	0.005

*Values are no./total (%) or median (interquartile range). Pregnancy outcome was missing for 9 patients. CS, congenital syphilis. †CS cases include newborns with CS diagnoses, miscarriages, and stillbirths. ‡One pregnant woman delivered twins. The twin delivery was acknowledged as 1 birth outcome, and the birth weight of the first child born was used for the table and analyses. §Student *t*-test for continuous variables and either χ^2^ test or Fisher exact test for categorical variables. ¶Denominators varied because of missing data. Anywhere from 1 to 11 cases might be missing.

**Table 2:** Characteristics and birth outcomes of pregnant women with active syphilis treated with amoxicillin or ampicillin, by syphilis stage, Japan, 2010–2018*

**Category**	**Early syphilis, n = 31**	**Late syphilis, n = 49**	**p value†**
Age, y	26 (21–28)	25 (22–27)	0.63
Japanese	29/31 (94)	46/49 (94)	1.00
HIV co-infection	0/31	1/49 (2)	1.00
Diagnosis and treatment			
Rapid plasma reagin titer at diagnosis	44 (27–64)	58 (29–83)	0.64
Amoxicillin	28/31 (90)	38/49 (78)	0.23
Ampicillin	3/31 (10)	11/49 (22)	
Antimicrobial drug dosage, mg/d	1,500 (1,500–1,500)	1,500 (1,500–1,500)	0.56
Received 1,500 mg/d	26/31 (84)	35/49 (71)	0.22
Total duration of treatment, d‡	30 (28–64)	78 (51–104)	0.004
Duration of treatment at delivery, d‡	30 (26–56)	70 (29-98)	0.016
Gestational age at diagnosis, wk‡	15.1 (11.1–27.5)	13.3 (12–25.6)	0.70
Gestational age at treatment, wk‡	17.3 (13.3–28.7)	14.7 (13–26)	0.62
Gestational age at delivery, wk‡	39.4 (38.1–40.6)	38.9 (37.7–40.5)	0.10
Time from treatment to delivery, wk‡	15.7 (0.4–25.9)	23 (16.7–27.2)	0.73
Birth outcomes			
Live birth with congenital syphilis diagnosis	0/26	13/45 (29)	0.009
Stillbirth	0/26	1/45 (2)	
Miscarriage	0/26	1/45 (2)	
Birth weight, g‡	2,902 (2,652–3,184)	2,942 (2,555–3,155)	0.88
Low birth weight, <2,500 g‡	3/26 (12)	8/45 (18)	0.52
Very low birth weight, <1,500 g‡	0/26	3/45 (7)	0.29

*Values are no./total (%) or median (interquartile range). †Used the Student *t*-test for continuous variables and either the χ^2^ test or Fisher exact test for categorical variables. ‡Denominators varied because of missing data. Anywhere from 1 to 11 cases might be missing.

Of the 80 cases, we excluded 9 (6 with unknown outcomes, 3 involving abortions induced at 15–17 weeks of pregnancy) from the outcome analysis. Of the remaining 71 cases, 15 (21%, 95% CI 13.2%–32%) were classified as CS (13 live newborns with CS diagnoses, 1 miscarriage, 1 stillbirth; Tables 2, 3; Appendix Table 1). Effectiveness of treatment for preventing CS cases was significantly better among patients with early syphilis than late syphilis; CS cases developed in 0% (0/26, 95% CI 0%–12.9%) of patients with early syphilis and 33% (15/45, 95% CI 21.4%–47.9%; p<0.001) of patients with late syphilis.

**Table 3:** Birth outcomes of pregnant women with active syphilis treated with oral amoxicillin or ampicillin, Japan, 2010–2018*

Category	Total no.	No. missing	No. live births without CS diagnosis	No. adverse outcomes			Adverse outcomes, % (95% CI)	p value†
				Live births with CS diagnosis	Miscarriages	Stillbirths		
All patients	80	9	56	13	1	1	21.1 (13.2–32)	
Early syphilis	31	5	26	0	0	0	0 (0–12.9)	<0.001
Late syphilis	49	4	30	13	1	1	33.3 (21.4–47.9)	
Amoxicillin	66	8	47	9	1	1	19.0 (10.9–30.9)	0.19
Ampicillin	14	1	9	4	0	0	30.8 (12.7–57.6)	
Japanese	75	9	54	10	1	1	18.2 (10.7–29.1)	0.033
Non-Japanese	5	0	2	3	0	0	60.0 (23.1–88.2)	
Patients starting syphilis treatment >60 days before delivery‡								
All	57	1	48	8	0	0	14.2 (7.4–25.7)	
Early syphilis	21	0	21	0	0	0	0 (0–15.5)	0.017
Late syphilis	36	1	27	8	0	0	22.9(12.1–39)	
Amoxicillin	46	1	40	5	0	0	11.1 (4.8–23.5)	0.11
Ampicillin	11	0	8	3	0	0	27.3 (9.7–56.6)	
Japanese	54	1	47	6	0	0	11.3 (5.3–22.6)	0.026
Non-Japanese	3	0	1	2	0	0	66.7 (20.8–93.9)	
Patients starting syphilis treatment <60 days before delivery								
All	14	0	8	5	0	1	42.9 (21.4–67.4)	
Early syphilis	5	0	5	0	0	0	0 (0–43.5)	0.028
Late syphilis	9	0	3	5	0	1	66.7 (35.4–87.9)	
Amoxicillin	13	0	8	4	0	1	38.5 (17.7–64.5)	0.43
Ampicillin	1	0	0	1	0	0	100 (20.7–100)	
Japanese	12	0	7	4	0	1	41.7 (19.3–68.1)	0.86
Non-Japanese	2	0	1	1	0	0	50.0 (9.5–90.6)	

*Nine cases (including 3 cases of induced abortion) were excluded from this analysis because data on birth outcomes were not available. One miscarriage case is missing in the subgroup analysis because the exact date of miscarriage was unknown, CS, congenital syphilis. †Used either the χ^2^ test or Fisher exact test to measure the association between cases of live birth without CS diagnosis and cases of live birth with CS diagnosis, miscarriage, and stillbirth. ‡Outcome data for 1 patient is missing. Outcome data (whether CS or not) was not known because this patient was referred to another hospital before delivery. Because the birth date of this patient’s infant was reported and available, we could group this patient with the appropriate group, which was the group that started syphilis treatment >60 days before delivery.

Among pregnant women with early syphilis, 26 (84%) received 1,500 mg/day of either amoxicillin or ampicillin, and the median duration of antimicrobial drug treatment was 30 (IQR 28–64) days (Table 2). Among those with late syphilis, 35 (71%) received 1,500 mg/day of either amoxicillin or ampicillin, and the median duration of antimicrobial drug treatment was 78 (IQR 51–104) days. CS cases developed in 19% (11/58, 95% CI 10.9%–30.9%) of those treated with amoxicillin and 31% (4/13, 95% CI 12.7%–57.6%; p = 0.19) of those treated with ampicillin (Table 3). CS cases were frequently found among non-Japanese pregnant women (60% [3/5], 95% CI 23.1%–88.2%).

In the subgroup of women initiating syphilis treatment >60 days before delivery, 14% (8/56 95% CI 7.4%–25.7%) had pregnancies resulting in CS cases (Table 3). In the subgroup initiating treatment <60 days before delivery, 43% (6/14, 95% CI 21.4%–67.4%) had pregnancies resulting in CS. The proportions of women with early and late syphilis were not different between these 2 treatment initiation subgroups (p = 0.57 by χ^2^ test). Among those who initiated syphilis treatment >60 days before delivery, 11% (5/45, 95% CI 4.8%–23.5%) of those treated with amoxicillin and 27% (3/11, 95% CI 9.7%–56.6%; p = 0.11) of those treated with ampicillin had pregnancies resulting in CS cases.

Because no CS cases developed among pregnant women with early syphilis, we applied exact logistic regression (*27*) to estimate exact odds ratios (ORs) and 95% CIs associated with various factors. Analyses showed late syphilis was associated with CS cases (late vs. early syphilis, adjusted exact OR 13.5, 95% CI 2.56–∞; p = 0.0025) and that starting treatment >60 days before delivery had a protective effect (>60 days vs. <60 days, adjusted exact OR 0.11, 95% CI 0–0.69; p = 0.023) (Appendix Table 2).

Discontinuation of antimicrobial drug treatment occurred because of adverse events in 3 (3.8%) of 80 pregnant women. All 3 started amoxicillin and switched to other antimicrobial drugs because of skin rash only, itching only, or dizziness only; 1 of 3 of these patients had a newborn with a CS diagnosis. 

All 15 women who had CS pregnancy outcomes had latent syphilis of unknown duration (Appendix Table 1). The 13 newborns with CS diagnoses had the following findings: FTA-ABS test results positive for serum IgM (n = 10), positive serum RPR titers (n = 5), CSF findings (n = 3), very low (<1,500 g; n = 2) or extremely low (<1,000 g; n = 1) birth weight, clinical signs compatible with CS (n = 2), and cardiac anomaly (n = 1). (Appendix Table 1).

A 4-fold decrement in serum RPR titer was achieved in 35 (49%) pregnant women by delivery, 41 (53%) at 6 months after treatment, and 46 (82%) at 1 year after treatment (Table 4). The percentage of CS cases was not significantly different between pregnant women with (16%, n = 5) and without (23%, n = 8) a 4-fold titer decline at delivery (p = 0.54). However, RPR titers declined >4-fold in 91% (21/23) of women with early syphilis (group with 0 CS cases) and 76% (25/33) of women with late syphilis (group with 15 CS cases) (Table 4). The percentage who achieved a 4-fold decrement was higher among those with early syphilis than among those with late syphilis for all 3 endpoints (by delivery, 58% early vs. 44% late; 6 months, 67% early vs. 45% late; 1 year, 91% early vs. 76% late). Among those who started treatment >60 days before delivery, 63% (35/56) had late syphilis, and 81% (35/43) achieved a 4-fold RPR titer decline by 1 year after treatment (Tables 3, 4).

**Table 4:** Serologic outcomes of pregnant women with active syphilis treated with amoxicillin or ampicillin, Japan, 2010–2018, by delivery, 6 months after treatment, and 1 year after treatment*

Patient group	No./total (%) with >4-fold decrement in RPR titer			
	By delivery	6 months after treatment	1 year after treatment	
All	35/71 (49)	41/77 (53)	46/56 (82)	
Live birth without CS diagnosis	27/54 (50)	29/54 (54)	31/39 (80)	
CS cases†	5/13 (39)	6/15 (40)	9/11 (82)	
Early syphilis	15/26 (58)	20/30 (67)	21/23 (91)	
Late syphilis	20/45 (44)	21/47 (45)	25/33 (76)	
Amoxicillin	27/58 (47)	32/63 (51)	36/44 (82)	
Ampicillin	8/13 (62)	9/14 (64)	10/12 (83)	
Japanese	34/66 (52)	39/72 (54)	44/52 (85)	
Non-Japanese	1/5 (20)	2/5 (40)	2/4 (50)	
Started syphilis treatment >60 days before delivery	32/55 (58)	32/55 (58)	35/43 (81)	
Started syphilis treatment <60 days before delivery	0	5/17 (29)	6/8 (75)	

*CS, congenital syphilis; RPR, rapid plasma reagin. †CS cases include newborns with CS diagnoses, miscarriages, and stillbirths.

## Discussion

In this nationwide, multicenter retrospective study, we evaluated effectiveness and tolerability of oral amoxicillin or ampicillin for treatment of pregnant women with active syphilis and prevention of MTCT of syphilis. Overall, 21% (15/71) of pregnancies resulted in CS cases, which we defined to include CS diagnoses in live newborns, miscarriages, and stillbirths. Among pregnancies in women who initiated syphilis treatment >60 days before delivery, 14% (8/56) resulted in CS cases. However, no CS cases were observed among the subgroup with early syphilis (n = 26), suggesting that oral amoxicillin with 1,500-mg dosing (500 mg 3×/d) for 30 days could be an optional regimen for treatment of pregnant women with early syphilis when intramuscular BPG is not available. In this study, we found a high percentage of CS cases (33%, 15/45) among those with late syphilis treated with oral amoxicillin or ampicillin, suggesting these regimens should not be used in this population. Late initiation of treatment was also associated with CS independent of syphilis stage. Although further studies evaluating drug adherence are warranted before any recommendation can be made, these findings are valuable for implicating some alternative treatments, considering reports of syphilis increases among heterosexual populations, reports of CS increases in various countries (including Japan and the United States), and national shortages of BPG (*28*,*29*).

Our study has 3 strengths. First, we evaluated effectiveness of oral amoxicillin or ampicillin for the treatment of pregnant women with active syphilis and prevention of MTCT. The finding that 1,500 mg of oral amoxicillin for 30 days might be effective in treating pregnant women with early syphilis and preventing CS is clinically useful because intramuscular BPG, the only recommended regimen for this population, is not always available because of shortages or stockouts (*3*,*9*). High-dose oral amoxicillin (3,000 mg/d) plus probenecid has been reported to effectively treat syphilis in nonpregnant patients with HIV infection with a serologic effectiveness of 97.5% for early syphilis and 90.8% for late syphilis (*10*). Further studies are warranted to determine efficacy, optimal dosing, duration, and the need for co-administration of probenecid with oral amoxicillin as an alternative regimen for both early and late syphilis in pregnant women.

Second, the well-established and strict inclusion criteria of having both serum RPR titers >8 and positive treponemal test results, regardless of symptoms, for active syphilis was a strength of this study (*10*,*15*,*19*). Application of these criteria enabled us to exclude those with low RPR titers who might have had previous antimicrobial drug treatment or those with serofast status, for whom further treatment is not required (*30*). This inclusion criterion was necessary to exclude persons who had past (not current) infections, considering that various antimicrobial drugs can treat syphilis (*30*,*31*) and that patients can receive effective treatment for syphilis without recognizing it.

Third, we evaluated not only MTCT of syphilis but also treatment effectiveness of oral amoxicillin or ampicillin in women using serology. Serologic effectiveness 1 year after treatment was 82% overall and 91% among early syphilis cases, suggesting greater effectiveness of this regimen for early syphilis. Furthermore, as was the case for the report by Rac et al. (*25*), we showed that the percentage of CS cases was not different between patients with and without a 4-fold titer decline at delivery.

The effectiveness of antimicrobial drugs for the prevention of MTCT of syphilis needs to be carefully considered because evidence is limited and diagnostic criteria for pregnant women with syphilis and CS vary across studies (*5*,*8*,*25*). We included time between treatment and delivery and syphilis staging in analyses because these parameters influence MTCT; transmission is more likely to occur during early syphilis stages and among women with late treatment (*30*,*32*). The CDC-recommended regimen of intramuscular BPG once for early syphilis and three times at 1-week intervals for late syphilis was reported to prevent 98.2% of CS cases (97.1% for early syphilis, 100% for late syphilis including latent syphilis of unknown duration) among a cohort of pregnant women with syphilis diagnosed during 1987–1989 (*5*). However, note that the authors of that investigation applied loose inclusion criteria (positive for treponemal antibody and positive by either the venereal disease research laboratory test or the RPR test); their diagnostic criteria for CS was strict (*5*). That group of authors subsequently reported that, among a cohort of pregnant women with syphilis treated during 1981–2011 by CDC guidelines, 18% of infants subsequently required treatment for CS (*25*). Only 1 case series has been published on the use of ceftriaxone to treat pregnant women with syphilis and to prevent CS (*8*). In that study, 2 courses of intramuscular ceftriaxone with 250-mg dosing for 7–10 days was evaluated in 11 pregnant women with early syphilis, and this treatment resulted in no CS diagnoses.

Our study demonstrates the need for comparative trials to evaluate the use of amoxicillin in the prevention of MTCT of syphilis before recommending this regimen as an alternative to BPG for pregnant women with syphilis. Considering our study results, healthcare authorities in Japan and other countries where intramuscular BPG is not recommended should consider making intramuscular BPG available. When intramuscular BPG is not available to treat pregnant women with syphilis, physicians need to make difficult decisions regarding alternative regimens, often following recommendations for neurosyphilis, such as intravenous aqueous crystalline penicillin G or intravenous or intramuscular ceftriaxone, treatments with limited clinical evidence available on efficacy.

We found that 17% of pregnant women were treated with oral ampicillin, even though oral amoxicillin is favored over oral ampicillin for most indications because of greater bioavailability (*31*,*33*). Oral ampicillin is also used to prevent group B *Streptococcus* at some facilities in Japan, despite guidelines recommending the injectable form of ampicillin for such indications (*11*,*34*). Our study results showed that the effectiveness of oral amoxicillin and ampicillin were not significantly different; however, our sample size was small.

Our study has several limitations. First, the retrospective nature of the study could have introduced some selection and information bias, and variables were missing for some mothers and infants. Second, data on adherence of patients to the prescribed regimens were not available, could not be evaluated, and could have affected our findings. Third, the definition of CS is not universal; diagnostic criteria can differ in national surveillance guidelines of other countries, limiting the comparativeness of our findings with other studies (*4*,*12*,*22*). In this study, we followed the reporting criteria set by the Japanese Ministry of Health, Labour, and Welfare, which includes the criterion of FTA-ABS test positivity for serum IgM in newborns (*12*,*20*). In contrast to IgG, IgM does not cross the placenta, making IgM in the serum of newborns a reliable marker with high specificity for CS diagnosis (*21*,*35*). However, a sufficiently sensitive and specific IgM assay is not commercially available (*4*). CDC guidelines do not include FTA-ABS test positivity for serum IgM in newborns as a criterion for CS diagnosis (*4*), whereas the Japan and the European Centre for Disease Prevention and Control guidelines do (*12*,*20*,*22*). In our study, misclassification of infants with CS could have been possible because evaluation (including laboratory testing) of newborns was not always rigorously performed. CS can cause long bone and CSF findings; these criteria are used in the United States for CS diagnosis (*4*). Because long bone radiographs are not included in the reporting criteria for CS in Japan, these procedures are infrequently performed, and results were not available for review in this analysis. Among 71 cases with available birth outcomes, 60 had serum RPR test results and only 6 had CSF results (3 with remarkable findings). Fourth, even though we classified latent syphilis of unknown duration as late syphilis, this category could have included early latent syphilis; clearly distinguishing between these 2 groups is difficult. Last, our results should be interpreted with caution because of the small sample size.

In conclusion, we evaluated the effectiveness and tolerability of oral amoxicillin and ampicillin for the treatment of pregnant women with active syphilis and prevention of MTCT of syphilis in Japan. Although we cannot recommend oral amoxicillin or ampicillin as alternative regimens for the treatment of pregnant women with syphilis, this analysis suggests that 1,500 mg/day of oral amoxicillin for 30 days could effectively treat early syphilis and prevent CS. Further studies, preferably controlled comparative trials, are needed to establish the efficacy of oral amoxicillin as an alternative regimen for the treatment of pregnant women with syphilis and the prevention of CS.

Appendix Additional information about effectiveness and tolerability of oral amoxicillin in pregnant women with active syphilis, Japan, 2010–2018.

## References

[1] Korenromp EL, Rowley J, Alonso M, Mello MB, Wijesooriya NS, Mahiané SG, et al. Global burden of maternal and congenital syphilis and associated adverse birth outcomes-Estimates for 2016 and progress since 2012. PLoS One. 2019;14:e0211720. 10.1371/journal.pone.0211720 30811406 PMC6392238

[2] Lawn JE, Blencowe H, Waiswa P, Amouzou A, Mathers C, Hogan D, et al.; Lancet Ending Preventable Stillbirths Series study group; Lancet Stillbirth Epidemiology investigator group. Stillbirths: rates, risk factors, and acceleration towards 2030. Lancet. 2016;387:587–603. 10.1016/S0140-6736(15)00837-5 26794078

[3] World Health Organization. WHO guidelines for the treatment of *Treponema pallidum* (syphilis). 2016 [cited 2020 Jan 30]. http://apps.who.int/iris/bitstream/10665/249572/1/9789241549806-eng.pdf 27631046

[4] Workowski KA, Bolan GA; Centers for Disease Control and Prevention. Sexually transmitted diseases treatment guidelines, 2015. MMWR Recomm Rep. 2015;64(RR-03):1–137. 26042815 PMC5885289

[5] Alexander JM, Sheffield JS, Sanchez PJ, Mayfield J, Wendel GD Jr. Efficacy of treatment for syphilis in pregnancy. Obstet Gynecol. 1999;93:5–8. 9916946 10.1016/s0029-7844(98)00338-x

[6] Roberts CP, Raich A, Stafylis C, Klausner JD. Alternative treatments for syphilis during pregnancy. Sex Transm Dis. 2019;46:637–40. 10.1097/OLQ.0000000000001050 31517802 PMC6750027

[7] Molini BJ, Tantalo LC, Sahi SK, Rodriguez VI, Brandt SL, Fernandez MC, et al. Macrolide resistance in *Treponema pallidum* correlates with 23S rDNA mutations in recently isolated clinical strains. Sex Transm Dis. 2016;43:579–83. 10.1097/OLQ.0000000000000486 27513385 PMC4982755

[8] Zhou P, Gu Z, Xu J, Wang X, Liao K. A study evaluating ceftriaxone as a treatment agent for primary and secondary syphilis in pregnancy. Sex Transm Dis. 2005;32:495–8. 10.1097/01.olq.0000170443.70739.cd 16041252

[9] Nurse-Findlay S, Taylor MM, Savage M, Mello MB, Saliyou S, Lavayen M, et al. Shortages of benzathine penicillin for prevention of mother-to-child transmission of syphilis: An evaluation from multi-country surveys and stakeholder interviews. PLoS Med. 2017;14:e1002473. 10.1371/journal.pmed.1002473 29281619 PMC5744908

[10] Tanizaki R, Nishijima T, Aoki T, Teruya K, Kikuchi Y, Oka S, et al. High-dose oral amoxicillin plus probenecid is highly effective for syphilis in patients with HIV infection. Clin Infect Dis. 2015;61:177–83. 10.1093/cid/civ270 25829004

[11] Japan Society of Obstetrics and Gynecology; Japan Association of Obstetricians and Gynecologists. The Japanese obstetrics and gynecology care guidelines [in Japanese]. 2017 [cited 2020 Jan 30]. http://www.jsog.or.jp/activity/pdf/gl_fujinka_2017.pdf

[12] Japanese Society for Sexually Transmitted Infections. The Japanese STI diagnosis and treatment guidelines [in Japanese]. 2016 [cited 2020 Jan 30]. http://jssti.umin.jp/pdf/guideline-2016_v2.pdf

[13] Katanami Y, Hashimoto T, Takaya S, Yamamoto K, Kutsuna S, Takeshita N, et al. Amoxicillin and ceftriaxone as treatment alternatives to penicillin for maternal syphilis. Emerg Infect Dis. 2017;23:827–9. 10.3201/eid2305.161936 28418316 PMC5403051

[14] Takamatsu K, Kitawaki J. Annual report of the Women’s Health Care Committee, Japan Society of Obstetrics and Gynecology, 2017. J Obstet Gynaecol Res. 2018;44:13–26. 10.1111/jog.13470 29082573

[15] Kenyon C, Lynen L, Florence E, Caluwaerts S, Vandenbruaene M, Apers L, et al. Syphilis reinfections pose problems for syphilis diagnosis in Antwerp, Belgium - 1992 to 2012. Euro Surveill. 2014;19:20958. 10.2807/1560-7917.ES2014.19.45.20958 25411690

[16] Marra CM, Maxwell CL, Smith SL, Lukehart SA, Rompalo AM, Eaton M, et al. Cerebrospinal fluid abnormalities in patients with syphilis: association with clinical and laboratory features. J Infect Dis. 2004;189:369–76. 10.1086/381227 14745693

[17] Zetola NM, Engelman J, Jensen TP, Klausner JD. Syphilis in the United States: an update for clinicians with an emphasis on HIV coinfection. Mayo Clin Proc. 2007;82:1091–102. 10.4065/82.9.1091 17803877

[18] Lee JH, Lim CS, Lee MG, Kim HS. Comparison of an automated rapid plasma reagin (RPR) test with the conventional RPR card test in syphilis testing. BMJ Open. 2014;4:e005664. 10.1136/bmjopen-2014-005664 25552608 PMC4281540

[19] Tsuboi M, Nishijima T, Aoki T, Teruya K, Kikuchi Y, Gatanaga H, et al. Usefulness of automated latex turbidimetric rapid plasma reagin test for diagnosis and evaluation of treatment response in syphilis in comparison with manual card test: a prospective cohort study. J Clin Microbiol. 2018;56:e01003-18. 10.1128/JCM.01003-18 30135229 PMC6204675

[20] Government of Japan. Case report form of congenital syphilis [in Japanese] [cited 2020 Jan 30]. https://www.mhlw.go.jp/bunya/kenkou/kekkaku-kansenshou11/pdf/01-05-11-3-b.pdf

[21] Herremans T, Kortbeek L, Notermans DW. A review of diagnostic tests for congenital syphilis in newborns. Eur J Clin Microbiol Infect Dis. 2010;29:495–501. 10.1007/s10096-010-0900-8 20336337

[22] European Union. Decisions. Commission implementing decision (EU) 2018/945 of 22 June 2018 on the communicable diseases and related special health issues to be covered by epidemiological surveillance as well as relevant case definitions. Off J Eur Union. 2018;61:L170/44–5 [cited 2020 Jan 30] https://eur-lex.europa.eu/legal-content/EN/TXT/PDF/?uri=OJ:L:2018:170:FULL&from

[23] Cheng JQ, Zhou H, Hong FC, Zhang D, Zhang YJ, Pan P, et al. Syphilis screening and intervention in 500,000 pregnant women in Shenzhen, the People’s Republic of China. Sex Transm Infect. 2007;83:347–50. 10.1136/sti.2006.023655 17693449 PMC2659022

[24] Janier M, Hegyi V, Dupin N, Unemo M, Tiplica GS, Potočnik M, et al. 2014 European guideline on the management of syphilis. J Eur Acad Dermatol Venereol. 2014;28:1581–93. 10.1111/jdv.12734 25348878

[25] Rac MW, Bryant SN, Cantey JB, McIntire DD, Wendel GD Jr, Sheffield JS. Maternal titers after adequate syphilotherapy during pregnancy. Clin Infect Dis. 2015;60:686–90. 10.1093/cid/ciu920 25414264

[26] World Health Organization. Global guidance on criteria and processes for validation: elimination of mother-to-child transmission of HIV and syphilis. 2nd edition. 2017 [cited 2020 Jan 30]. https://www.who.int/reproductivehealth/publications/emtct-hiv-syphilis

[27] Mehta CR, Patel NR. Exact logistic regression: theory and examples. Stat Med. 1995;14:2143–60. 10.1002/sim.4780141908 8552893

[28] Takahashi T, Arima Y, Yamagishi T, Nishiki S, Kanai M, Ishikane M, et al. Rapid increase in reports of syphilis associated with men who have sex with women and women who have sex with men, Japan, 2012 to 2016. Sex Transm Dis. 2018;45:139–43. 10.1097/OLQ.0000000000000768 29420439 PMC5815645

[29] Centers for Disease Control and Prevention, National Center for HIV/AIDS, Viral Hepatitis, STD, and TB Prevention. Sexually transmitted disease surveillance 2017. 2018 Sep [cited 2019 Jun 10]. https://www.cdc.gov/std/stats17/2017-STD-Surveillance-Report_CDC-clearance-9.10.18.pdf

[30] Clement ME, Okeke NL, Hicks CB. Treatment of syphilis: a systematic review. JAMA. 2014;312:1905–17. 10.1001/jama.2014.13259 25387188 PMC6690208

[31] Bennett J, Dolin R, Blaser MJ. Mandell, Douglas, and Bennett’s principles and practice of infectious diseases. 8th edition. London: Churchill Livingstone Elsevier; 2014

[32] Sheffield JS, Sánchez PJ, Morris G, Maberry M, Zeray F, McIntire DD, et al. Congenital syphilis after maternal treatment for syphilis during pregnancy. Am J Obstet Gynecol. 2002;186:569–73. 10.1067/mob.2002.121541 11904625

[33] Klein JO, Finland M. The new penicillins. N Engl J Med. 1963;269:1129–34. 10.1056/NEJM196311212692107 14052279

[34] AmericanCollege of Obstetricians and Gynecologists Committee on Obstetric Practice. Prevention of group B streptococcal early-onset disease in newborns: ACOG committee opinion summary, number 797. Obstet Gynecol. 2020;135:e51–72. 10.1097/AOG.0000000000003668 31977793

[35] Johnston NA. Neonatal congenital syphilis. Diagnosis by the absorbed fluorescent treponemal antibody (IgM) test. Br J Vener Dis. 1972;48:464–9. 10.1136/sti.48.6.464 4568466 PMC1048368

