# Supplementary material for: Effectiveness and Tolerability of Oral Amoxicillin in Pregnant Women with Active Syphilis, Japan, 2010–2018
Source: Emerg Infect Dis. 2020 Jun;26(6):1192–200. doi: 10.3201/eid2606.191300 (PMC7258477; doi:10.3201/eid2606.191300)
Supplement: Appendix — Additional information about effectiveness and tolerability of oral amoxicillin in pregnant women with active syphilis, Japan, 2010–2018. [file 19-1300-Techapp-s1.pdf]

Article DOI: https://doi.org/10.3201/eid2606.191300

Effectiveness and Tolerability of Oral Amoxicillin in Pregnant Women with Active Syphilis, Japan, 2010–2018

Appendix

Study Setting and Population

Participating institutions were selected based on the participation in the previous study on mother-to-child transmission (MTCT) and perinatal abnormality due to sexually transmitted infection (STI) in 2016 conducted by the Women's Health Care Committee of Japan Society of Obstetrics and Gynecology (JSOG) which invited 628 core hospitals across Japan, including university hospitals, tertiary care hospitals, and teaching hospitals, and were joined by 257 hospitals (*1*). Among 257 facilities, 88 facilities which reported syphilitic pregnant women were invited for the participation to the present study and the protocol and the Case Report Form were sent for collection of data.

Data Collection

Data on the following parameters were collected with the Case Report Form (CRF) from the medical chart at each facility for pregnant women with syphilis: date of birth, race, date of syphilis diagnosis, treatment and delivery, gestational weeks at syphilis diagnosis, serum rapid plasma reagin (RPR) titer at diagnosis and all available RPR titers and their dates within one year after treatment, serum treponemal test at diagnosis, stage of syphilis infection, symptoms of syphilis, syphilis treatment regimen and dosing and treatment duration, concurrent use of probenecid, switch of antimicrobial drugs due to adverse events and detailed information on adverse events, HIV coinfection, and birth outcome, such as live birth, still birth or miscarriage. For a newborn, body weight, serum RPR titer and treponemal test and their dates, serum fluorescent treponemal antibody absorption (FTA-ABS) IgM, other relevant diagnostic test, and diagnosis of congenital syphilis (CS) and rationale for the diagnosis were collected.

Appendix Table 1. Characteristics of 15 congenital syphilis cases, including cases with congenital syphilis diagnosis, stillbirth and miscarriage*

Age of pregnant women (year)	Maternal syphilis stage	Race	Gestational age at treatment initiation	Baseline serum RPR titer	Antibiotics	Antibiotic dosage (mg/d)/duration by delivery (days)	Antibiotic duration for pregnant women (days)	Gestational age at delivery	Birth weight (g)	Birth outcome	Rationale for CS diagnosis	Serum RPR titer of newborn	CSF of newborn
29	Latent syphilis with unknown duration	Japanese	41w 6d	55.0	Amoxicillin	1,000/4	60	42w 3d	1,145	CS live birth	Very low birth weight, hepatosplenomegaly, thrombocytopenia	NA	NA
22	Latent syphilis with unknown duration	Japanese	12w 2d	225.0	Ampicillin	1,500/167	167	38w 1d	3,120	CS live birth	Positive FTA-ABS IgM	–	NA
23	Latent syphilis with unknown duration	Japanese	12w 4d	105.0	Ampicillin	1,500/70	70	38w 4d	2,518	CS live birth	Positive FTA-ABS IgM	–	NA
17	Latent syphilis with unknown duration	Thai	36w	125.0	Amoxicillin	1,500/34	101	40w 6d	3,050	CS live birth	Positive FTA-ABS IgM, abnormal CSF results	4.6	FTA-ABS 25, TPLA 3,395
27	Latent syphilis with unknown duration	Japanese	34w 2d	28.0	Amoxicillin	1,500/6	27	35w 1d	2,524	Stillbirth	Not applicable	NA	NA
23	Latent syphilis with unknown duration	Japanese	29w 3d	91.4	Amoxicillin	1,500/2	33	29w 5d	1,214	CS live birth	Very low birth weight, positive FTA-ABS IgM, abnormal CSF results	5.6	FTA-ABS 4
26	Latent syphilis with unknown duration	Vietnamese	13w 4d	32.0	Ampicillin	1,500/106	106	38w 4d	3,500	CS live birth	Positive FTA-ABS IgM	–	NA
19	Latent syphilis with unknown duration	Japanese	14w	15.4	Amoxicillin	1,500/98	98	44w	2,845	CS live birth	Positive FTA-ABS IgM	–	NA
20	Latent syphilis with unknown duration	Peruvian	18w 1d	16.0	Amoxicillin	1,500/93	93	NA	NA	CS	Cardiac anomaly	NA	NA
25	Latent syphilis with unknown duration	Japanese	24w 6d	256.0	Amoxicillin	1,500/66	66	40w 4d	2,704	CS live birth	Positive FTA-ABS IgM	–	NA

Age of pregnant women (year)	Maternal syphilis stage	Race	Gestational age at treatment initiation	Baseline serum RPR titer	Antibiotics	Antibiotic dosage (mg/d)/duration by delivery (days)	Antibiotic duration for pregnant women (days)	Gestational age at delivery	Birth weight (g)	Birth outcome	Rationale for CS diagnosis	Serum RPR titer of newborn	CSF of newborn
26	Latent syphilis with unknown duration	Japanese	13w 4d	64.0	Amoxicillin	1,500/84	84	40w	3,140	CS live birth	Positive FTA-ABS IgM	4	NA
41	Latent syphilis with unknown duration	Japanese	9w	64.0	Amoxicillin	3,000/NA	28	NA	NA	Miscarriage	Not applicable	NA	NA
22	Latent syphilis with unknown duration	Japanese	34w	58.0	Ampicillin	1,500/1	37	34w	976	CS live birth	Extremely low birth weight, hearing loss suspected	135	NA
26	Latent syphilis with unknown duration	Japanese	36w	28.0	Amoxicillin	1,500/1	57	36w	2,380	CS live birth	$\geq$4 fold serum RPR titer of newborn than the mother, positive FTA-ABS IgM, abnormal CSF results	1220	Positive RPR, positive TPHA
24	Latent syphilis with unknown duration	Japanese	18w 2d	34.5	Amoxicillin	1,500/105	105	41w	2,936	CS live birth	Positive FTA-ABS IgM	–	NA

*In Japan, the automated latex turbidimetric immunoassay for the RPR test has been widely used, in addition to the conventional manual RPR card test. The automated RPR test is highly correlated to the manual test for the diagnosis of syphilis. Thus the present study treats the titer of automated RPR test as the same value as that of manual RPR test. CS, congenital syphilis; CSF, cerebrospinal fluid; FTA-ABS, fluorescent treponemal antibody-absorption; NA, not available; RPR, rapid plasma reagin; TPHA, *Treponema pallidum* hemagglutination; TPLA, *Treponema pallidum* latex-agglutination; –, negative result.

Appendix Table 2. Exact multivariate logistic regression analysis: association between various factors and congenital syphilis cases (n = 69)

Category	Adjusted exact odds ratio (95% CI)	P value
Late syphilis versus early syphilis	13.5 (2.56-infinity)	0.0025
Starting treatment at least 60 days before delivery versus starting treatment <60 days before delivery	0.11 (0-0.69)	0.023
Duration of treatment >30 days versus <30 days	2.40 (0.41-infinity)	0.23
Amoxicillin versus ampicillin	1.23 (0.20-7.19)	1.00

Reference

1. Takamatsu K, Kitawaki J. Annual report of the Women's Health Care Committee, Japan Society of Obstetrics and Gynecology, 2017. J Obstet Gynaecol Res. 2018;44:13-26.

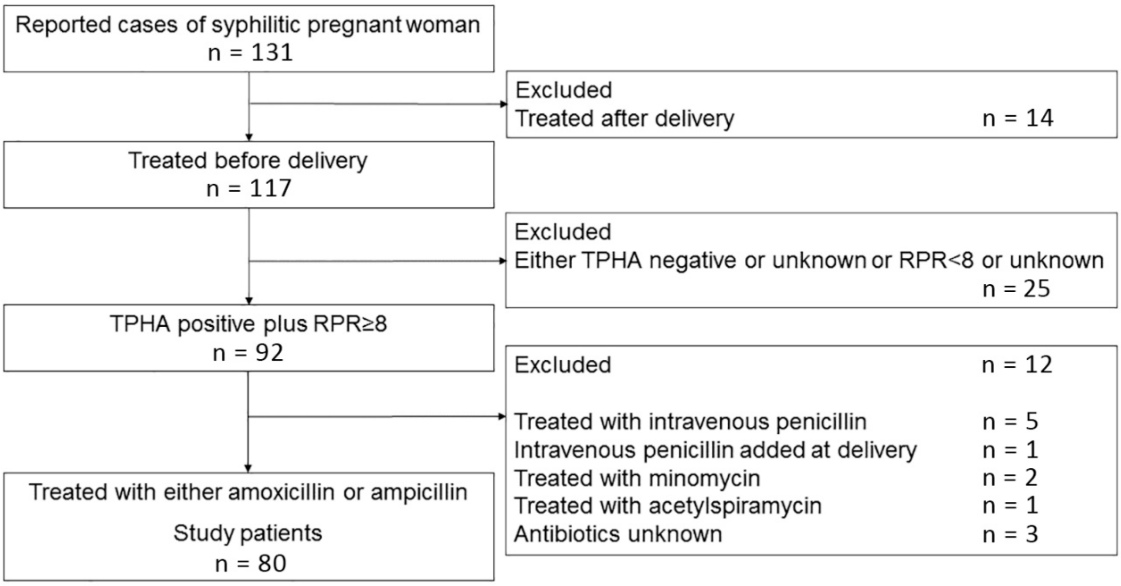

Appendix Figure. Patient inclusion. RPR, rapid plasma reagin; TPHA, *Treponema pallidum* hemagglutination.
